# Flower Extracts of the Invasive Species *Prunus serotina* Ehrh.: Phytochemical Composition, Protective Effects on Human Plasma Components, and Impact on Neutrophil Functions

**DOI:** 10.3390/ijms27198558

**Published:** 2026-09-24

**Authors:** Anna Magiera, Anna Marchelak, Joanna Kołodziejczyk-Czepas, Aleksandra Dowgird, Monika Anna Olszewska

**Affiliations:** 1 Department of Pharmacognosy, Faculty of Pharmacy, Medical University of Lodz, 1 Muszynskiego St., 90-151 Lodz, Poland; anna.marchelak@umed.lodz.pl (A.M.); monika.olszewska@umed.lodz.pl (M.A.O.); 2 Department of General Biochemistry, Faculty of Biology and Environmental Protection, University of Lodz, 141/143 Pomorska St., 90-236 Lodz, Poland; joanna.kolodziejczyk@biol.uni.lodz.pl; 3 Student Scientific Association, Department of Pharmacognosy, Faculty of Pharmacy, Medical University of Lodz, 1 Muszynskiego St., 90-151 Lodz, Poland; dowgird.aleksandra@gmail.com

**Keywords:** black cherry, Rosaceae, flower extract, phenolic compounds, neutrophils, anti-inflammatory, antioxidant, cardiovascular diseases

## Abstract

*Prunus serotina* Ehrh. (Rosaceae) is one of the most widespread invasive tree species in Europe. Its abundant floral biomass remains understudied, despite its potential as a source of valuable bioactive compounds. Our study provides a comprehensive phytochemical characterization of *P. serotina* flower extracts and the first evaluation of their biological activity in models related to inflammation and oxidative stress. Phytochemical composition was analyzed using UHPLC-PDA-ESI-MS^3^, HPLC-PDA, and spectrophotometric techniques. Over 60 specialized metabolites were fully or partially identified, primarily polyphenolic compounds (flavonols, phenolic acids, proanthocyanidins), and the content of major constituents was quantified using authentic reference standards, providing the most detailed profiling data on *P. serotina* flowers to date. The total phenolic content of the extracts, obtained using water, 70% ethanol–water, ethyl acetate, and *n*-butanol, ranged from 119.05 to 144.97 mg/g dw. Biological potential of the extracts was evaluated by assessing their effects on pro-inflammatory cytokine release (TNF-α), the inflammation-related enzyme elastase (ELA-2), and reactive oxygen species (ROS) level in stimulated human immune cells (neutrophils), as well as their ability to enhance plasma antioxidant capacity (NEAC) and protect human plasma proteins and lipids from peroxynitrite-induced damage. At concentrations of 1–100 µg/mL, the extracts decreased TNF-α, ELA-2, and ROS levels by up to 46%, 43%, and 74%, respectively, and reduced 3-nitrotyrosine by up to 72%. These findings highlight the potential of *P. serotina* biomass as a sustainable source of bioactive compounds for inflammation- and oxidative stress-related disorders, particularly cardiovascular diseases (CVDs). However, further in vivo studies are needed to verify these effects under physiological conditions and assess their pharmacological potential.

## 1. Introduction

*Prunus serotina* Ehrh., commonly known as black cherry (rum cherry or mountain black cherry), is a member of the Rosaceae family. Although native to the United States, black cherry is now found on nearly every continent [1,2,3,4]. It was introduced to Europe in the early 17th century by France for the production of commercially valuable timber and has been cultivated in Germany since the late 18th century. At present, *P. serotina* is distributed from Spain to Ukraine and from Scandinavian Peninsula to Italy. It is considered one of the most invasive and problematic woody species in Europe. The species is notable for its capacity to adapt to adverse environmental conditions. Black cherry threatens European ecosystems and biodiversity by occupying habitats of native trees such as hornbeams, beeches, and oaks, thereby disrupting local flora. Additionally, *P. serotina* displaces native *Prunus* species, including *Prunus padus* L. (bird cherry) [4,5,6,7]. As a result, significant efforts are being made to control and eradicate this species. Control strategies include mechanical approaches, such as felling, and chemical methods, such as the application of herbicides. Mechanical interventions generate waste biomass consisting of wood, leaves, bark, and reproductive organs, including flowers. Frequently, this material is not utilized for any valuable purpose, and the removal of the invasive species incurs forest management costs. Nevertheless, this biomass has potential for purposeful use, which could partially offset the expenses associated with species control. On the other hand, the complete eradication of black cherry from European ecosystems using reasonable measures and costs is, in practice, extremely challenging, if not impossible. Therefore, exploring alternative uses for this species is warranted [5,8].

The phytotherapeutic potential of many of black cherry organs remains insufficiently explored [1,3]. In its native range, *P. serotina* bark/branches have been used in traditional medicine to prepare syrups, infusions, and tinctures with sedative, tonic, astringent, anti-diabetic, and antitussive effects. The fruit is also used in the traditional medicine of North and Central Americas for its laxative properties when fresh and constipating effects when dried. Sirup from fruit is commonly used to treat coughs and respiratory conditions such as bronchitis and asthma. In traditional Mexican medicine, the fruit has been used to address gastrointestinal disorders, upper respiratory tract infections, hypertension, cardiovascular conditions, and diabetes. Seeds are used in decoction for the treatment of cough. Current ethnopharmacological studies do not document medicinal uses of flowers themselves [1,3,9,10]. However, given the anti-inflammatory, antioxidant, vasoprotective, and cardioprotective properties of polyphenolic phytochemicals (flavonol glycosides, flavone glycosides, phenolic acids, proanthocyanidins) found in the flowers of Rosaceae species such as *Crataegus laevigata* (Poir.) DC., *Crataegus monogyna* Jacq. [11,12], *Prunus padus* L. [13], *Prunus spinosa* L. [14,15,16] or *Sorbus aucuparia* L. [17], it is reasonable to expect that *P. serotina* flowers may also contain biologically active polyphenolic secondary metabolites.

Data on the polyphenolic composition of black cherry flowers are sparse but promising, as the plant material has been shown to contain flavonol glycosides (derivatives of quercetin, kaempferol, and isorhamnetin), flavones, and flavanones. Preliminary compositional studies have also demonstrated the presence of phenolic acids (caffeoylquinic, feruloylquinic, and coumaroylquinic acids) and B-type proanthocyanidins in the plant material [18,19,20,21]. Hitherto, there are no studies on the biological activity of *P. serotina* flowers [1,3].

In this study, we investigated *P. serotina* flower extracts by evaluating their **(1)** qualitative and quantitative phytochemical profiles using UHPLC-PDA-ESI-MS^3^, HPLC-PDA, and spectrophotometric methods; **(2)** protective effects on human plasma proteins and lipids under peroxynitrite (ONOO^–^) induced oxidative stress; **(3)** impact on plasma total antioxidant capacity; **(4)** influence on the selected proinflammatory and prooxidant functions of human neutrophils; and **(5)** ability to scavenge in vivo relevant reactive oxygen species (ROS), including H_2_O_2_, O_2_^•−^, and HO^•^. Given the important role of chronic inflammation and oxidative stress in processes associated with endothelial dysfunction, atherosclerosis, and thrombosis, the present complementary in vitro and ex vivo studies were designed to provide a preliminary assessment of the potential of *P. serotina* flowers as a source of bioactive compounds with antioxidant and anti-inflammatory properties that may have pharmacological relevance to inflammation- and oxidative stress-related disorders, particularly cardiovascular diseases (CVDs). Although these effects were observed under experimental rather than physiological conditions, the findings provide a basis for further investigation of their pharmacological potential. These studies align with current scientific priorities, such as sustainable resource use and the search for new biologically active natural materials.

## 2. Results and Discussion

### 2.1. Polyphenolic Compounds

According to the collected knowledge, *P. serotina* flowers are characterized by the presence of various polyphenols; however, phytochemical studies conducted so far have focused mainly on the dominant constituents of the flowers, resulting in limited information about the complete polyphenolic profiles of the plant material [18,19,20,21]. In our study, we prepared extracts using various solvents as, due to the distinct physicochemical properties of individual polyphenolic groups, their effective isolation from plant material requires the use of extractants of varying polarity. The current literature suggests that the most effective extractants for polyphenols are alcohol–water or acetone–water mixtures, as well as ethyl acetate, *n*-butanol, and water. To ensure exhaustive and selective extraction for the identification of polyphenolic compounds from the plant material, the following solvents were employed: 70% ethanol–water mixture, water, ethyl acetate, and *n*-butanol.

A total of 71 peaks corresponding to secondary metabolites in black cherry flowers were detected in the analyzed extracts during the LC-MS study; of these, 65 were assigned identities based on comprehensive data analysis (Table 1, Figure 1). Fifteen compounds were confirmed by comparing their retention times and UV-Vis and MS/MS spectral characteristics with those of authentic reference standards. The remaining compounds were assigned tentative identities based on the agreement of their *m*/*z* values and characteristic fragment ions with the published literature data. When analytical data allowed determination of the general structural nature but did not permit unambiguous elucidation of the complete structure, the compounds were described at the appropriate structural level. This approach was particularly relevant when it was not possible to distinguish between structural or positional isomers, or to determine the identity, sequence, or linkage of sugar units and the precise site of glycosylation. Similarly, for dimeric compounds—such as proanthocyanidins—and various phenolic acid derivatives, annotations were limited to the level supported by information derived from the available mass spectrometry data. In these cases, compounds were assigned to an appropriate structural type (such as dihexoside or proanthocyanidin dimer), or a potential isomer, without specifying details that could not be reliably determined using the employed analytical methods.

Eleven compounds (peaks **13**, **15**, **16**, **20**, **25**, **29**, **33**, **39**, **41**, **44**, and **53**) belonging to the flavan-3-ol group were identified in the black cherry flower extracts [22,23]. The newly detected flavan-3-ol derivatives included procyanidin B1 (**13**), procyanidin B2 (**25**), procyanidin C1 (**39**), and gallocatechin/epigallocatechin (**15**). It should also be noted that a total of eight B-type procyanidin dimers were detected among the flavan-3-ol derivatives; previous study indicated the presence of only two B-type procyanidin dimers [18]. In the group of phenolic acids, 29 compounds were detected in *P. serotina* flowers (compounds **1–3**, **5–7**, **9–12**, **14**, **17–19**, **22–24**, **26–28**, **31**, **32**, **34–38**, **42**, **60**) [24,25,26,27,28,29,30,31]. The newly recognized phenolic acids in the flower samples were quinic acid (**1**) and its derivative (**2**), hydroxybenzoic acid derivative (**5**), two caffeic acid 3/4-*O*-hexosides (**12**, **23**) and one other derivative (**3**), two *O*-dihexosides of coumaric acid (**6**, **14**) and two other derivatives (**10**, **24**), protocatechuic acid hexoside (**7**), vanillic acid hexoside (**18**), cryptochlorogenic acid (**22**), two isomers of caffeoylferuloylquinic acid (**28**, **34**), 3/4-*O*-caffeoylshikimic acid (**32**), *p*-coumaric acid (**35**), 4-*O*-*p*-feruloylquinic acid (**37**), 4-*O*-*p*-coumaroylquinic acid (**31**), and acetyl-4-*O*-*p*-coumaroylquinic acid (**42**). Additionally, a total of 18 flavonols were identified in the plant material studied—derivatives of quercetin (compounds **40**, **49–52**, **54**, **57**, **62**, **65**, **68**), kaempferol (compounds **47**, **55**, **63**, **66**), and isorhamnetin (structures **43**, **46**, **58**, **59**) [32]. Comparing the results obtained in our study with the previous literature reports [18,19,20,21], we can conclude that an additional kaempferol hexoside (**55/66**), quercetin dihexoside (**40**), and quercetin rhamnopyranoside (**65**) were detected in *P. serotina* flowers. The presence of four flavanone compounds—derivatives of eriodictyol, naringenin, and pinocembrin (structures **30**, **48**, **56**, **61**) [18,33]—was also detected in the plant material. Three compounds—prunin (naringenin 7-*O*-β-glucoside; **56**), pinocembrin hexoside (**61**), and one eriodictyol hexoside (**30/48**)—were identified for the first time in black cherry flowers. The terpenoid roseoside (**21**) was also newly identified in the flowers as a formate adduct [34]. Phenylamide compounds, specifically spermidine derivatives [17,35], were detected for the first time as well (peaks **69**, **71**). The number of detected compounds was similar across solvents, ranging from 43 to 49.

**Table 1:** UHPLC-PDA-ESI-MS^3^ identification data of compounds detected in the extracts from flowers of *P. serotina*.

No	Compound	R_t_ (min)	UV_Λmax_ (nm)	[M–H]^−^ (*m*/*z*)	Fragmentary Ions	Extract				References
						Ethanol–Water	Water	Ethyl Acetate	*n*-Butanol	
**1**	Qunic acid	1.4	264 285	191	-	+	+	+	+	[25]
**2**	Qunic acid derivative	1.5	265 285	533	191, 173	+	−	+	+	[25]
**3**	Caffeic acid derivative	2.1	272	275	179, 161	−	+	−	−	-
**4**	Unidentified	2.3	260	311	265	+	−	+	−	-
**5**	Hydroxybenzoic acid derivative	2.5	295	367	137	−	−	−	+	-
**6**	Coumaric acid *O*-dihexoside I	3.9	252 290	487	325, 163	+	−	−	−	[24,28]
**7**	Protocatechuic acid hexoside	4.5	300	315	153	+	+	−	+	[28]
**8**	Unidentified	4.8	272	203	-	−	+	−	−	-
**9**	Dicaffeoylquinic acid	6.0	279	515	353, 191	+	+	−	−	-
**10**	Coumaric acid derivative	6.6	300	393	163	+	+	+	+	-
**11**	Coumaric acid hexoside (formate adduct)	6.7	295	371	325, 163	+	+	+	+	[30]
**12**	Caffeic acid 3/4-*O*-hexoside I	7.3	300 325	341	179, 161, 135	+	+	+	+	-
**13**	Procyanidin B1 ^A^	9.1	280 312	577	559, 451, 425, 407, 289	+	+	−	−	-
**14**	Coumaric acid *O*-dihexoside II	9.2	315	487	325, 163	−	−	+	+	[24,28]
**15**	Gallocatechin/epigallocatechin	9.4	312	305	245	−	−	+	−	[23]
**16**	Procyanidin type B dimer I	9.7	280 315	577	559, 451, 425, 289	+	+	−	−	[22]
**17**	Coumaric acid *O*-hexoside	9.8	285	325	163	−	−	+	+	-
**18**	Vanillic acid hexoside	10.1	230 280	329	167	+	−	+	−	-
**19**	5-*O*-Caffeoylquinic acid (chlorogenic acid) ^A^	10.5	295 325	353	191	+	+	+	+	-
**20**	Procyanidin type B dimer II	11.2	289 325	577	425, 407, 289	−	+	−	−	[22]
**21**	Roseoside (formate adduct)	11.3	296 318	431	385, 161	+	+	+	+	[34]
**22**	4-*O*-Caffeoylquinic acid (cryptochlorogenic acid) ^A^	11.7	290 325	353	173, 191	+	+	+	+	-
**23**	Caffeic acid 3/4-*O*-hexoside II	12.2	328	341	179, 161	+	+	+	+	-
**24**	*p*-coumaric acid derivative	13.1	323	387	369, 341, 207, 163	−	+	+	+	[26]
**25**	Procyanidin B2 ^A^	13.2	280 330	577	425, 407, 289	+	−	−	−	-
**26**	*cis*-3/5-Caffeoylquinic acid	13.3	320	353	191	−	−	−	+	-
**27**	5-*O*-*p*-Coumaroylquinic acid	14.0	216 311	337	191, 163	+	+	+	+	[27]
**28**	Caffeoylferuloylquinic acid isomer I	14.3	286 312	529	485, 161	−	−	+	−	[29]
**29**	Epicatechin ^A^	14.3	280 315	289	-	+	+	−	−	-
**30**	Eriodictyol hexoside I	14.5	290 313	449	287	−	−	+	+	[18]
**31**	4-*O*-*p*-Coumaroylquinic acid	14.7	294 313	337	173	+	+	+	+	[27]
**32**	3/4-*O*-Caffeoylshikimic acid	15.0	293 324	335	179, 135	+	+	+	+	[27]
**33**	Procyanidin type B dimer III	15.3	280 323	577	289	+	−	−	−	[22]
**34**	Caffeoylferuloylquinic acid isomer II	15.6	310	529	349, 161, 117	−	−	+	−	[29]
**35**	*p*-Coumaric acid ^A^	15.6	307	163	-	−	+	−	−	-
**36**	5-*O*-*p*-Feruloylquinic acid	15.7	292 325	367	191, 173	+	−	−	+	[31]
**37**	4-*O*-*p*-Feruloylquinic acid	16.1	290 320	367	191, 173	−	+	+	+	[31]
**38**	Coumaroylquinic acid isomer	17.0	300	337	191	+	+	+	+	[31]
**39**	Procyanidin C1 ^A^	17.5	280 314	865	577, 407, 377, 289	+	+	−	−	-
**40**	Quercetin dihexoside	18.6	265 355	625	463, 301	−	−	−	+	-
**41**	Procyanidin type B dimer IV	18.7	272	577	425, 407, 289	+	+	−	−	[22]
**42**	Acetyl-4-*O*-*p*-coumaroylquinic acid	18.7	223 279 322	379	337, 173	−	−	+	−	[31]
**43**	Isorhamnetin derivative	19.9	270 342	785	623, 315	−	−	−	+	-
**44**	Procyanidin type B dimer V	20.4	278	577	289	+	+	−	−	[22]
**45**	Unidentified	20.6	280	381	335, 161	−	−	+	−	-
**46**	Isorhamnetin dihexoside	21.7	269	639	477, 459, 315	+	+	+	+	-
**47**	Kaempferol dihexoside	23.1	265 340	609	447, 285	+	−	−	−	-
**48**	Eriodictyol hexoside II	23.8	280	449	287	+	+	+	+	[18]
**49**	Quercetin rhamnoside-hexoside	24.9	350	609	301	+	+	+	+	-
**50**	Quercetin 3-*O*-β-D-galactoside (hyperoside) ^A^	25.8	256 352	463	301	+	+	+	+	-
**51**	Quercetin 3-*O*-(6″-*O*-α-L-rhamnopyranosyl)-β-D-glucopyranoside (rutin) ^A^	26.0	254 350	609	301	+	+	+	+	-
**52**	Quercetin 3-*O*-β-D-glucopyranoside (isoquercetrin) ^A^	27.1	265 350	463	301	+	−	+	+	-
**53**	Procyanidin type B dimer VI	28.2	280	577	425, 407, 289	+	+	−	−	[22]
**54**	Quercetin 3-*O*-α-L-xylopyranoside (reinutrin) ^A^	29.3	255 352	433	301	+	+	+	+	-
**55**	Kaempferol hexoside	31.4	264 346	447	285	+	+	+	+	-
**56**	Naringenin 7-*O*-β-glucoside (prunin) ^A^	32.8	345	433	271	−	−	+	−	-
**57**	Quercetin 3-*O*-α-L-arabinofuranoside (avicularin) ^A^	34.3	256 351	433	301	+	+	+	+	-
**58**	Isorhamnetin hexoside	35.8	264 354	477	315	+	+	+	+	[32]
**59**	Isorhamnetin 3-*O*-(6″-*O*-α-L-rhamnopyranosyl)-β-D-glucopyranoside (narcissin) ^A^	36.4	269 356	623	315	+	+	+	+	-
**60**	Dicaffeoylquinic acid	37.8	325	515	353, 191	+	+	+	+	-
**61**	Pinocembrin hexoside	38.6	264 344	417	255	+	+	+	+	[33]
**62**	Quercetin acetyl-hexoside	39.8	320	505	463, 301	+	+	+	+	-
**63**	Kaempferol 3-*O*-α-L-arabinofuranoside (juglanin) ^A^	41.6	264 345	417	285	+	+	+	+	-
**64**	Unidentified	43.7	256 353	475	301	−	−	+	−	-
**65**	Quercetin rhamnopyranoside	44.1	345	447	301	−	−	+	+	-
**66**	Kaempferol hexoside	43.9	280 320	447	285	+	−	−	−	-
**67**	Unidentified	47.0	255 353	475	301	−	−	+	−	-
**68**	Quercetin rhamnoside-hexoside	49.6	314 363	609	463, 301	+	−	+	−	-
**69**	Dicaffeoyl-coumaroyl-spermidine isomer	51.6	295 316	614	452, 358	+	+	+	+	[35]
**70**	Unidentified	53.4	289 311	597	451, 351, 287	+	+	+	+	-
**71**	Tricoumaroyl-spermidine isomer	54.5	292 310	582	462, 342	+	+	+	+	[17]

^A^ Identified with authentic standards. Rt, retention times. UV λmax, absorbance maxima in UV-Vis spectra. [M–H]^−^, deprotonated molecular ions in MS spectra recorded in a negative ion mode; +, present; −, absent.

No cyanogenic glycosides were detected in *P. serotina* flower extracts using the applied LC-MS method. This finding is important, as cyanogenic glycosides have previously been reported or suggested to occur in certain *Prunus* species and specific plant tissues, particularly the bark, seeds, and fruits [36,37,38]. The presence of these compounds is a significant safety concern, since exposure to cyanogenic compounds from plants has been linked to adverse health effects in humans, including neurological disorders or other chronic health complications [36]. Consequently, evaluating the potential occurrence of these compounds is essential when considering plant biomass for biomedical applications. The absence of cyanogenic glycosides in the current LC-MS analysis suggests a favorable safety profile for the analyzed plant material. However, this result alone does not establish the overall safety of *P. serotina* flowers. Additional targeted phytochemical analyses and biological studies with appropriate models are needed to fully assess their safety of use.

Among the prepared extracts, the 70% ethanol–water extract demonstrated the highest total polyphenol content (TPC) that amounted to 144.97 mg/g dw, as measured by the Folin–Ciocalteu method. The other extracts showed comparable TPC values, ranging from 119.05 to 124.28 mg/g dw (Table 2), which were 14.3–17.5% lower than those measured in the ethanol–water extract. The ethanol–water extract also showed the highest total proanthocyanidin content (TPAC) of 81.08 mg/g dw, as determined with the *n*-butanol-HCl spectrophotometric method. The aqueous extract had a relatively high TPAC of 46.37 mg/g dw, though this was significantly lower than that of the ethanol–water extract. In contrast, the ethyl acetate and *n*-butanol extracts exhibited low TPAC values, amounting to 6.16 and 6.72 mg/g dw, respectively (Table 2). Proanthocyanidins (flavan-3-ol derivatives) constituted 55.9%, 39.0%, 5.12%, and 5.41% of the TPC levels in the ethanol–water, water, ethyl acetate, and *n*-butanol extracts, respectively. HPLC analysis indicated that most proanthocyanidins were high-molecular-weight compounds. The content of low-molecular-weight flavan-3-ols (monomers, dimers, trimers; TFAC) was 9.04 mg/g in the ethanol–water extract and 4.95 mg/g dw in the water extract; these compounds were not detected in the other extracts. The predominant compounds in this group were epicatechin (2.63–4.07 mg/g dw) and procyanidin B1 (1.44–1.97 mg/g dw). All extracts contained high flavonol contents (TFC). TFC values for the 70% ethanol–water and water extracts were similar, ranging from 49.72 to 54.97 mg/g dw, while the ethyl acetate and *n*-butanol extracts both had TFC values of ~ 89–90.0 mg/g dw (Table 2). Flavanols represented 58.2%, 59.9%, 91.9%, and 81.8% of all low-molecular-weight polyphenols (TLPC) quantified in the ethanol–water, water, ethyl acetate, and *n*-butanol extracts, respectively. Among the quercetin derivatives, hyperoside (quercetin 3-*O*-β-D-galactoside) predominated, with concentrations ranging from 20.60 to 39.26 mg/g dw across extracts. Avicularin (quercetin 3-*O*-α-L-arabinofuranoside), another quercetin derivative, was also abundant, with levels between 10.58 and 28.33 mg/g dw. For isorhamnetin derivatives, the dihexoside form was dominant, with amounts from 5.30 to 6.24 mg/g dw in all the extracts, except for the ethyl acetate extract, where this compound was present in traces, below the quantification limit. Among kaempferol derivatives, kaempferol hexoside (4.02–10.96 mg/g dw) and juglanin (kaempferol 3-*O*-α-L-arabinofuranoside; 2.10–9.44 mg/g dw) were present in significant amounts. Regarding phenolic acids, the ethanol–water and water extracts had the highest and comparable total contents of phenolic acids (TPHAC), ranging from 27.64 to 29.44 mg/g dw, while the ethyl acetate extract revealed the lowest TPHAC at 7.97 mg/g dw. Chlorogenic acid was the main phenolic acid, reaching particularly high concentrations in the ethanol–water and water extracts (23.96–25.44 mg/g dw), compared to 5.33 mg/g dw in the ethyl acetate extract and 15.74 mg/g dw in the *n*-butanol extract (Table 2). Phenolic acids accounted for 31.2%, 33.3%, 8.10%, and 18.2% of TLPC in the analyzed extracts. Because only a single extraction process was performed for each solvent, interpret observed differences in individual compounds or compound groups as differences between the specific extracts analyzed in this study.

### 2.2. Biological Activity

Oxidative stress arises from an imbalance between the generation and elimination of ROS (i.a. H_2_O_2_, O_2_^•−^, HO^•.^, and ONOO^−^). Oxidative stress responses are implicated in numerous cellular and tissue processes that contribute to the development of CVDs. All major cardiovascular risk factors, including hypercholesterolemia, hypertension, diabetes, poor diet, and smoking, are linked to increased ROS production. ROS influence critical processes such as vascular smooth muscle cell proliferation and migration, nonenzymatic eicosanoid production from arachidonic acid, arterial vasoconstriction, and platelet aggregation. Moreover, ROS induce structural modifications of functional proteins and lipids in the blood plasma, including nitration and dimerization of tyrosine units, or lipid peroxidation, which ultimately leads to chronic cardiovascular dysfunction [39,40,41]. Additionally, above-mentioned risk factors stimulate the infiltration into the endothelium of inflammatory cells, such as neutrophils or monocytes, which serve as further sources of ROS. In addition, azurophilic granules of neutrophils harbor various serine proteases, including neutrophil elastase (ELA-2), which degrades a tissue inhibitor pathway or promoting coagulation. Evidence also suggests that ELA-2 can cleave and modify certain low-density lipoproteins (LDLs), thereby significantly increasing their uptake by arterial wall cells (particularly macrophages), leading to the accumulation of cellular cholesterol. Furthermore, this enzyme degrades elastin (an extracellular matrix protein), causing damage to blood vessels and increasing their permeability [42,43]. Immune cells are a major source of proinflammatory cytokines, including tumor necrosis factor alpha (TNF-α), which is central to chronic inflammation in CVDs. TNF-α activates endothelial cells and increases the expression of adhesion molecules, thereby facilitating inflammatory cell recruitment and atherosclerotic lesion progression. Persistently elevated TNF-α levels are associated with an increased risk of plaque destabilization and cardiovascular events [44,45].

During the initial phase of activity studies, the ROS (H_2_O_2_, O_2_^•−^, and HO^•^) scavenging capacities of all four extracts were evaluated using cell-free models. Both O_2_^•^− and HO^•^ are critical in initiating and amplifying the oxidative stress cascade, causing damage to lipids, proteins, and disruption in cellular function in CVDs. O_2_^•−^ is central in the early stages, as it transforms into more reactive molecules. Superoxide dismutase (SOD) converts O_2_^•−^ to H_2_O_2_, which, although less reactive, diffuses easily across membranes and acts as a substrate for further oxidative reactions. In the presence of Fe^2+^ or Cu^+^, H_2_O_2_ undergoes the Fenton reaction to form the highly reactive HO^•^. This radical initiates lipid peroxidation by abstracting hydrogen from polyunsaturated fatty acids, producing lipid peroxides. O_2_^•−^ can also react with NO to form ONOO^–^, a potent oxidizing and nitrating agent [46,47]. The tested ethanol–water and water extracts demonstrated notably strong O_2_^•−^ scavenging activity, with SC_50_ values between 13.0 and 16.6 µg/mL. These values were 9 to 11 times lower than the SC_50_ observed for Trolox^®^ (positive control; a water-soluble analog of vitamin E with strong antioxidant properties). Furthermore, the extracts exhibited comparable or only marginally reduced HO^•^ scavenging capacity relative to the positive control. The SC_50_ values for the extracts amounted to 121.1 and 145.2 µg/mL, whereas the SC_50_ for Trolox^®^ was 128.1 µg/mL. The ethyl acetate and *n*-butanol extracts showed higher SC_50_ values than the water and ethanol–water extracts in both assays. In the O_2_^•−^ scavenging assay, their activity was 1.4 to 3.6 times lower, and in the HO^•^ scavenging assay, the activity was 1.6 to 2.5 times lower. In the H_2_O_2_ scavenging assay, all four extracts were significantly less active than Trolox^®^, with SC_50_ values 6.2 to 8.5 times higher. The ethanol–water extract demonstrated the most potent H_2_O_2_ scavenging capacity, whereas the *n*-butanol extract exhibited the lowest activity (Table 3).

The results of the preliminary activity studies indicated that among the analyzed extracts, the ethanol–water and water extracts were the most effective scavengers of various ROS. These extracts exhibit a polyphenolic composition that closely mirrors the profile of bioactive compounds found in herbal preparations commonly used in traditional medicinal practice, i.e., tinctures and infusions. In contrast, ethyl acetate and *n*-butanol extracts represent only purified fractions of selected polyphenolic compounds. On these grounds, only the ethanol–water and water extracts were chosen for further biological activity studies in more advanced models.

In studies using cell models and human plasma, extract concentrations of 1, 5, 50, and 100 µg/mL were used. This corresponded to polyphenol contents, expressed as gallic acid equivalents (GAE), of 0.14, 0.72, 7.24, and 14.50 µg GAE/mL for the ethanol–water extract and 0.12, 0.60, 5.95, and 11.91 µg GAE/mL for the water extract, respectively. This conversion is an approximate indicator of the total phenolic content and may provide an indicative reference for the extent of exposure to polyphenolic compounds. However, the actual bioavailability and circulating concentrations of specific polyphenols may differ, depending on absorption, metabolism, conjugation, protein binding, or other pharmacokinetic processes.

In our studies, both the ethanol–water and water extracts increased plasma non-enzymatic antioxidant capacity (NEAC) across all tested concentrations (Figure 2A). NEAC serves as a reliable marker for evaluating the effectiveness of antioxidant interventions in preventing and treating vascular diseases. Higher NEAC values indicate greater plasma capacity to neutralize ROS, while reduced NEAC is frequently observed in individuals with risk factors such as smoking, obesity, or diabetes [48]. At a concentration of 50 µg/mL (5.95–7.24 µg GAE/mL), both extracts restored NEAC to baseline values observed prior to ONOO^–^ exposure. At concentrations of 1–5 µg/mL (0.12–0.72 µg GAE/mL), extracts increased NEAC by 26.1–41.3% compared to ONOO^–^-treated plasma. Across all tested concentrations, extracts demonstrated similar effectiveness to Trolox^®^ at 1–5 µg/mL. Studies also assessed the effect of extracts on plasma 3-nitrotyrosine (3-NT) levels (Figure 2B). 3-NT is an important marker of nitrative stress, which results from the modification of the amino acid tyrosine by reactive nitrogen species, particularly ONOO^–^. Elevated 3-NT levels are found in atherosclerosis and other conditions, indicating protein damage and vascular endothelial dysfunction. Determining 3-NT levels allows for the assessment of the degree of oxidative/nitrative damage and the effectiveness of antioxidant interventions [47,49]. The ethanol–water extract strongly reduced 3-NT levels across the entire range of tested concentrations. The decrease ranged from 57.9% to 72.2% at 1–5 µg/mL (0.14–0.72 µg GAE/mL) and 50 µg/mL (7.24 µg GAE/mL), respectively, being thus more pronounced or comparable to Trolox^®^ at the same concentrations (3-NT level reduction by 42.2% to 76.5%). The water extract, on the other hand, performed slightly less well than the positive control, but still reduced the level of the tested marker by 33.7–46.1%. The TBARS assay was employed as a measure of polyunsaturated fatty acid peroxidation in cell membranes and lipoproteins. Lipid peroxidation products accelerate atherosclerotic lesion development, generate additional free radicals, and perpetuate oxidative damage. They also activate immune cells and increase proinflammatory cytokine production [50,51]. The ethanol–water extract at concentrations of 5 and 50 µg/mL (0.72 and 7.24 µg GAE/mL) reduced TBARS levels by 67.5% compared to the stimulated control, demonstrating efficacy comparable to Trolox^®^ at the same concentrations (TBARS level reduction by 61.5–63.2%). The water extract was effective only at the highest concentration (50 µg/mL; 5.95 µg GAE/mL), resulting in a 49.8% reduction in TBARS levels (Figure 2C).

In the subsequent phase of the study, we evaluated the impact of *P. serotina* flower extracts on the secretion of three principal prooxidant and proinflammatory factors by human neutrophils. Oxidative stress is known to activate the immune system, resulting in increased neutrophil infiltration. Elevated neutrophil levels are linked to a greater risk and severity of CVDs and the neutrophil-to-lymphocyte ratio (NLR) serves as an important diagnostic and prognostic marker in CVDs [42,52]. Quercetin, a potent natural antioxidant and inhibitor of ELA-2, as well as dexamethasone, an anti-inflammatory agent, served as positive controls in the neutrophil studies (Figure 3A–C).

The extracts reduced ROS-associated chemiluminescence in stimulated human neutrophils. This may result from the extracts’ contribution to regulating cellular ROS generation. However, given the direct ROS-scavenging activity demonstrated for the extracts in cell-free assays, the observed reduction in chemiluminescence may also, at least in part, reflect direct scavenging of ROS generated by neutrophils. The aqueous-ethanolic extract reduced the ROS-associated chemiluminescence signal across all tested concentrations (5–100 µg/mL; corresponding to 0.72–14.50 µg GAE/mL), with the most pronounced effects observed at 50 and 100 µg/mL (7.24 and 14.50 µg GAE/mL, respectively), resulting in a 54.8–67.5% reduction in the chemiluminescence signal. The water extract at the same concentrations (5–100 µg/mL; 0.60–11.91 µg GAE/mL) reduced ROS levels by 41.0–73.9%. At concentrations of 50 and 100 µg/mL (5.95 and 11.91 µg GAE/mL), both extracts demonstrated a similar capacity to reduce ROS as quercetin at 5 and 50 µg/mL, achieving a 37.7–84.0% decrease (Figure 3A). In the ELA-2 assay, ethanol–water (5–100 µg/mL; 0.72–14.50 µg GAE/mL) and water (50–100 µg/mL; 5.95–11.91 µg GAE/mL) extracts reduced the pro-inflammatory enzyme by 18.0–42.9%. By comparison, quercetin (5–50 µg/mL) exhibited a moderately stronger effect, reducing ELA-2 by 55.0–63.1% (Figure 3B). The observed reduction in ELA-2 may result from both the inhibition of elastase release by neutrophils and the direct inhibition of its enzymatic activity by components of the extracts. At concentrations of 50 to 100 µg/mL (5.95–7.24 and 11.91–14.50 µg GAE/mL), both extracts markedly reduced TNF-α levels by 31.7–45.8%. The ethanol–water extract also produced a reduction in the secretion of this proinflammatory cytokine by 16.3% at a concentration of 5 µg/mL (0.72 µg GAE/mL). In comparison, dexamethasone (5–50 µg/mL) reduced TNF-α levels by 67.5–75.0% (Figure 3C). None of the tested extracts or positive controls reduced cell viability by more than 9.0%, as measured by flow cytometry. This minimal impact confirms that the observed reductions in mediator levels or production were not due to nonspecific effects from decreased cell viability.

The extensive scientific literature shows that plant polyphenols play a significant role in preventing and treating CVDs due to their diverse biological effects. Their broad pharmacological properties stem from complex mechanisms, including interference with enzymatic systems, cellular signaling pathways, functional proteins, and gene expression [53,54,55]. Polyphenols can directly reduce ROS and inhibit enzymes involved in ROS formation, such as xanthine oxidase, NADPH oxidase, myeloperoxidases, and nitric oxide synthase. Additionally, polyphenols can modify immune cell functions by influencing the secretion of proinflammatory cytokines and enzymes that remodel extracellular matrix components. A key molecular mechanism is their effect on nuclear transcription factors, primarily NF-κB, which regulates genes for proinflammatory cytokines, enzymes, acute phase proteins, and adhesion molecules, and NRF2, which controls the expression of antioxidant system proteins [53,56]. Our study evaluated selected antioxidant and anti-inflammatory properties of *P. serotina* flower extracts using complementary in vitro and ex vivo assays. In plasma studies, the observed protective effects suggest direct protection of plasma components against chemically induced oxidative stress. In the neutrophil model, the extracts modulated specific parameters of the cellular response, including ROS, TNF-α, and ELA-2 levels. A comprehensive analysis of molecular mechanisms potentially underlying these effects, such as activation of endogenous antioxidant mechanisms, regulation of signaling pathways, or gene expression, requires further verification.

Importantly, not all plant products rich in polyphenols demonstrate effective therapeutic outcomes, as their biological activity may depend on the presence and proportions of specific compounds. This report presents the first evidence that ethanol–water (7:3, *v*/*v*) and water extracts from the flowers of the invasive species *P. serotina* possess a complex polyphenolic composition with significant antioxidant and anti-inflammatory potential in both human neutrophil and human plasma models. It is noteworthy because the biological properties of this botanical raw material have not been previously investigated. The data expand the current understanding of the health-promoting potential of *Prunus* plants and reveal a previously unexplored source of bioactive compounds. As experiments with individual compounds or defined fractions were not performed in our study, the relative contribution of individual compounds or groups of compounds cannot be clearly distinguished. The observed activity may reflect the contribution of different classes of polyphenols, including flavan-3-ols, flavonols and phenolic acids. The additive or synergistic interactions between polyphenolic compounds are well documented in vitro and can significantly enhance anti-inflammatory and antioxidant properties [57,58].

The assumption that *P. serotina* flowers have health-promoting potential may stem from similarities between their phytochemical profile and those of other Rosaceae species. For example, our results align with previous findings on *P. spinosa* flowers, which also contain high levels of flavonols, proanthocyanidins, and phenolic acids. Extracts from these flowers demonstrate antioxidant, anti-inflammatory, and protective effects on plasma components, as well as regulation of hemostatic abnormalities, supporting their traditional use in cardiovascular therapy [14,15,16]. Likewise, flower preparations from *Crataegus* species are among the most well-documented phytotherapeutics for cardiovascular support. Their efficacy is attributed to the synergistic action of polyphenols, primarily proanthocyanidins and flavonols, which provide antioxidant, anti-inflammatory, and antihypertensive effects, regulate heart function, and enhance endothelial health [11,12]. The results indicate that the flowers of the studied species may serve as a promising alternative source of bioactive compounds compared to the more extensively researched Rosaceae flowers used in cardiovascular health preparations. Although this study examined selected biochemical and cellular endpoints rather than direct cardiovascular outcomes, these findings lay the groundwork for further investigation into the potential role of *P. serotina* flower extracts in supporting cardiovascular health. From a practical perspective, utilizing the biomass of invasive species for the extraction of bioactive compounds may be particularly advantageous.

Moreover, given the potential application of *P. serotina* flowers as a source of bioactive compounds, it is essential to evaluate their safety profile. The present study assessed the safety by examining the effects of extracts on human neutrophil viability. Across the tested concentration range of 5–100 µg/mL, no significant reduction in cell viability was detected, suggesting that the observed biological effects were not attributable to nonspecific cytotoxicity under the experimental conditions. Nevertheless, the absence of an effect on neutrophil viability does not constitute a comprehensive safety evaluation and precludes definitive conclusions regarding the in vivo safety of the extracts.

## 3. Materials and Methods

### 3.1. Plant Material and Extracts Preparation

Dried flowers of *Prunus serotina* Ehrh. were used in this study. The botanical identity of the plant material was confirmed by Dr. Anna Magiera from the Department of Pharmacognosy at the Medical University of Lodz. The material was collected in 2022 at the Rogów Arboretum of Warsaw University of Life Sciences (51°49′33″ N 19°53′20″ E). The plant material was collected from two areas and included flowers from multiple individual trees, sampled from different parts of each tree. After drying, the flowers were ground in an electric mill (ELDOM, Katowice, Poland) and sifted through a 0.315 mm sieve according to the pharmacopoeial guidelines for sample preparation of herbal medicinal substances [59]. Four extracts were prepared using ethanol–water (7:3, *v*/*v*), water, ethyl acetate, and *n*-butanol as extraction solvents. Prior to extraction, lipophilic components were removed by initial chloroform extraction. Three reflux cycles were then performed for exhaustive recovery of active components, and the resulting subextracts were combined. Each extraction cycle lasted 30 min and was performed at the solvent’s boiling point. The solvents were removed using the Rotavapor R-300 rotary evaporator (BÜCHI Labortechnik AG, Flawil, Switzerland) to yield dry extracts. The plant material used in this study was sourced from a single collection of *P. serotina* flowers and subjected to one extraction process. All tested extracts were prepared from this uniform batch of plant material. The dried extracts were stored in tightly closed amber glass vials at 4 °C, protected from light and moisture, until further analysis. Table 4 presents the extraction conditions and extract yields.

### 3.2. Identification of Polyphenolic Compounds by LC-PDA-ESI-MS^3^ Analyses

The qualitative profiling was conducted using the procedure described in our previous work [60]. The LC-PDA-ESI-MS^3^ analysis was performed on a UHPLC-3000 RS system (Dionex, Dreieich, Germany) coupled with a diode array detector (Dionex, Dreieich, Germany) and an AmaZon SL ion trap mass spectrometer with an ESI interface (Bruker Daltonik, Bremen, Germany). Separations were carried out on a Kinetex XB-C18 column (1.7 μm, 150 mm × 2.1 mm i.d.; Phenomenex, Torrance, CA, USA) at 25 °C. The mobile phase consisted of solvent A (water/formic acid, 100:0.1, *v*/*v*) and solvent B (acetonitrile) with the elution profile as follows: 0–10 min, 6–13% B (*v*/*v*); 10–15 min, 13% B (*v*/*v*); 15–19 min, 13–15% B (*v*/*v*); 19–28 min, 15% B (*v*/*v*); 28–44 min, 15–23% B (*v*/*v*); 44–55 min, 23–40% B (*v*/*v*); 55–60 min, 40% B (*v*/*v*); 60–65 min, 40–6% B (*v*/*v*); 65–80 min, 6% B (*v*/*v*). All chromatographic gradients were linear, and the flow rate was maintained at 0.3 mL/min. Prior to analysis, the extract solutions (16.0 mg/1.5 mL) were filtered using PTFE syringe filters (13 mm, 0.2 μm; Whatman, Pittsburgh, PA, USA). UV-Vis spectra were acquired in the range of 200–600 nm. The eluate was introduced directly into the ESI source without splitting and analyzed in the negative ion mode. The ESI source was operated with a nebulizer pressure of 40 psi, a dry gas flow rate of 9 L/min, a dry gas temperature of 300 °C, and a capillary voltage of 4.5 kV. For MS^2^/MS^3^ acquisition, the principal ion-isolation and fragmentation parameters were as follows: isolation width, 2 *m*/*z*; collision energy, 0.6–3.0 V; collision energy mode, gradient; fragmentation mode, SmartFrag; and fragmentation time, 40 ms. The injection volume was 3 µL. Mass spectra were recorded over an *m*/*z* range of 200–2200. All solvents were of HPLC grade (Avantor Performance Materials, Gliwice, Poland). Identification was confirmed by comparing retention times and UV-Vis and MS/MS spectra with reference standards when available. For compounds without available standards, preliminary identification relied on *m*/*z* values, characteristic fragmentation patterns, and UV-Vis spectral features. The resulting data for these compounds were compared with the published literature. A structure was tentatively assigned when the analytical parameters matched the characteristics of the proposed compound.

### 3.3. Quantitative HPLC Analysis

Phenolic compounds were quantitated using a Prominence-i HPLC system (Shimadzu, Kioto, Japan) equipped with a quaternary pump, column oven, autosampler, and photodiode array (PDA) detector. Chromatographic separations were performed at 20 °C using an Ascentis Express C18 column (2.7 µm, 150 × 4.6 mm; Supelco, Bellefonte, PA, USA) equipped with a guard column Ascentis^®^ Express C18, 2.7 Micron Guard Cartridge (2.7 μm, 5 mm × 4.6 mm; Supelco, Bellefonte, PA, USA). The mobile phase comprised 0.5% (*m*/*v*) orthophosphoric acid (solvent A) and acetonitrile (solvent B), both HPLC-grade (Avantor Performance). The gradient program was 0–1 min, 3% B in A (isocratic elution); 1–45 min, 3–35% B (linear gradient); 45–48 min, 35–50% B (linear gradient); 48–53 min, 50% B (isocratic elution); 53–55 min, 50–3% B (return to initial conditions); 55–60 min, 3% B (equilibration). The flow rate was 1.0 mL/min. The method was evaluated for selected analytical performance characteristics, including selectivity, calibration linearity, limits of detection and quantification (LOD and LOQ), and repeatability [61]. The evaluation was performed using 11 reference compounds, either purchased from PhytoLab GmbH & Co. KG (Vestenbergsgreuth, Germany) or isolated in-house, whose structures and purity had been verified by NMR [62,63,64]. Method selectivity was assessed by comparing the retention times and UV–vis spectra of analytes with those of the corresponding reference compounds. Linearity was evaluated by preparing a standard stock solution containing the 11 reference compounds in methanol–water (7:3, *v*/*v*), followed by serial dilution (in triplicate) with the same solvent to achieve at least five concentration levels (2%, 10%, 25%, 50%, 75%, and 100% of the stock concentration) within the range of 0.99–68.7 μg/mL. Samples were filtered through a PTFE syringe filter (25 mm, 0.22 µm, Vitrum) and injected in triplicate (5 μL) into the HPLC system. Calibration curve linearity was assessed using linear regression models (y = ax + b), and the statistical significance of the regression equations, slopes, and intercepts was determined using *F*-tests at the 99% confidence level. The limits of detection (LOD) and quantification (LOQ) were calculated from the standard deviation of the y-intercepts obtained from four independent calibration curves (σ) and the mean slope of these calibration curves (S), using LOD = 3.3σ/S and LOQ = 10σ/S, respectively. Repeatability was assessed using 6 independent determinations at 100% of the test concentration [58]. The evaluated parameters are summarized in Table 5. For sample analysis, three independent portions of each extract (approximately 17.5 mg each) were weighed and dissolved in methanol–water (7:3, *v*/*v*, 10 mL). Each sample preparation was filtered using a PTFE syringe filter (25 mm, 0.22 μm, Vitrum) and injected into the chromatographic system in triplicate (5 μL). Compound contents were calculated per dry weight (dw) of the extracts. The 11 compounds were quantified using calibration curves of their corresponding authentic reference standards. In contrast, the three tentatively identified compounds were estimated using calibration curves of selected reference standards and expressed as the corresponding reference-standard equivalents: isorhamnetin 3,7-di-*O*-β-D-glucopyranoside equivalents for isorhamnetin dihexoside, juglanin equivalents for kaempferol hexoside, and PB1 equivalents for proanthocyanidin B-type dimer.

### 3.4. Quantitative Profiling Using Spectrophotometric Methods

The total content of polyphenols (TPC) and condensed tannins (proanthocyanidins, TPAC) were quantified spectrophotometrically using the Folin–Ciocalteu and *n*-butanol-HCl assays, as previously described by Magiera et al. (2022) [60] and Olszewska et al. (2019) [17]. Results were reported as equivalents of HPLC-grade gallic acid (GAE) and procyanidin B2 (PB2) (Sigma-Aldrich, St. Louis, MO, USA) per dry weight (dw) of the extracts. For each extract, three independent sample preparations were analyzed, with five technical replicates per preparation.

### 3.5. Protective Potential on Human Plasma Components

Blood plasma was isolated from healthy volunteers using differential buffy coat centrifugation, as previously described [65,66]. Buffy coat fractions, which are byproducts of blood fractionation for transfusion, were obtained from the Regional Blood Donation and Blood Treatment Center in Łódź, Poland, and collected from adult donors over 18 years of age. All buffy coat units derived from blood collected from healthy donors eligible for blood donation and transfusion, according to applicable national regulations. Prior to distribution by the Regional Blood Donation and Blood Treatment Center, all units were fully anonymized. The conducted study did not involve the collection, processing, or storage of any personal data. The study was approved by the Committee on the Ethics of Research at the University of Lodz (8/KBBN-UŁ/II/2015).

For the assays, plasma samples were preincubated for 15 min at 37 °C with extracts or a positive control (Trolox^®^), then treated with ONOO^−^ (100–150 μM). Two control plasma samples were prepared: one exposed to ONOO^−^ without analytes, and a blank control containing neither analytes nor ONOO^−^. The protective effects of the tested analytes on human plasma components under ONOO^−^-induced oxidative stress were assessed spectrophotometrically and immunoenzymatically, as previously described [65,66].

The nonenzymatic antioxidant capacity (NEAC) of plasma was measured using the FRAP assay, based on the reduction in ferric ions (Fe^3+^) to ferrous ions (Fe^2+^). Fresh plasma samples were diluted with Ca^2+^-free PBS (0.02 M, pH 7.4) at a 1:4 volume ratio. The FRAP reagent was prepared from acetate buffer (300 mM, pH 3.6), TPTZ (10 mM in 0.04 M HCl), and ferric chloride (20 mM in 0.04 M HCl) at a volume ratio of 10:1:1, respectively. The diluted plasma samples were combined with the FRAP reagent in 96-well microplates. The standard curve was constructed using FeSO_4_ solution as the standard. After incubation for 15 min at 37 °C, FRAP values were measured at 593 nm using a Spectrostar Nano microplate spectrophotometer (BMG Labtech, Ortenberg, Germany) and reported as mM Fe^2+^ equivalents. Each sample was measured in duplicate.

A competitive ELISA was used for the immunodetection of 3-nitrotyrosine (3-NT) content. The procedure employed a 3-day protocol. On the first day, high-binding 96-well microtiter plates were coated with nitrated fibrinogen samples (1 μg/mL) and incubated overnight at 4 °C. On the following day, the wells were washed three times with 0.1 M Tris-HCl buffer (pH 9.0) containing 0.05% Tween 20 to remove unbound antigen. To minimize non-specific adsorption, the remaining binding sites were blocked with 1.5% defatted milk prepared in 0.1 M Tris-HCl buffer (pH 9.0), followed by incubation at 37 °C for 2 h. The wells were then washed three times with the same Tris-HCl/Tween buffer. For preparation of the antigen–antibody pre-incubation mixtures, plasma samples were diluted with 0.1 M Tris-HCl buffer (pH 9.0) at a 1:19 volume ratio. Anti-3-nitrotyrosine antibody solution was then added to the diluted plasma samples at a 1:1 volume ratio (dilution 1:20,000). The mixtures were incubated for 30 min at room temperature to allow interaction between free nitrated antigens present in the samples and the primary antibody. Subsequently, the pre-incubated sample-antibody mixtures were transferred to the antigen-coated wells. The plate was covered and incubated overnight at 4 °C. On the third day, the wells were washed three times with 0.1 M Tris-HCl buffer (pH 9.0) containing 0.05% Tween 20. Biotinylated secondary antibodies (dilution 1:5000) were added, and the plate was incubated for 1 h at room temperature. After three washing cycles with the same Tris-HCl/Tween buffer, streptavidin-horseradish peroxidase (HRP) conjugate solution (dilution 1:5000) was added, followed by incubation for 1 h at room temperature. Following another washing step, freshly prepared *O*-phenylenediamine (OPD) substrate solution was added, and color development was allowed to proceed for 10 min at room temperature. The reaction was then stopped by the addition of 40% sulfuric acid (H_2_SO_4_). A standard calibration curve was prepared using serial dilutions of a nitrated fibrinogen standard antigen, covering a range of 3-nitrotyrosine equivalent concentrations from 0.0114 to 2.94 μM. The calibration curve samples underwent the same protocol as the experimental samples. The content of 3-nitrotyrosine in the analyzed samples was quantified and expressed as equivalents of the nitrated standard antigen (3-NT-Fg). Absorbance was measured at 490 nm using a Spectrostar Nano microplate spectrophotometer (BMG Labtech, Ortenberg, Germany).

Plasma lipid peroxidation was measured using the thiobarbituric acid-reactive substances (TBARS) assay, and the results were expressed as nmol/mL of plasma. In the human plasma model assays, control samples were prepared from untreated plasma and/or plasma treated with ONOO^−^. Plasma samples were mixed with trichloroacetic acid (TCA) and thiobarbituric acid (TBA) at a 1:1:0.9 volume ratio, respectively. The reaction mixtures were vortexed vigorously for 1 min to disrupt the protein precipitate and subsequently shaken for 10 min to ensure thorough mixing. The samples were then incubated at 100 °C for 10 min using a BioSan CH3-150 Combitherm-2 heating-cooling block. Immediately after incubation, the tubes were transferred to an ice bath and cooled to room temperature. Following cooling, the samples were centrifuged at 13,000 rpm for 30 min at 20 °C using a Heraeus Biofuge Fresco centrifuge. The absorbance of the resulting supernatants was measured using a UV-Vis spectrophotometer (Thermo Fisher Scientific, Waltham, MA, USA).

Each experiment involving human plasma was performed using plasma isolated from several buffy coats derived from different donors. Hence, the letter “*n*” in the figure legends refers to the number of independent donors. To ensure technical reliability, each sample was analyzed in duplicate during both reaction development and visualization (two technical repeats were performed).

### 3.6. Bioactivity and Biocompatibility of Extracts in Human Neutrophil Models

Neutrophils were isolated from human buffy coats using dextran sedimentation, erythrocyte lysis, and Ficoll-Hypaque gradient centrifugation, as previously described [60]. Buffy coats were obtained from the Regional Blood Donation and Blood Treatment Center in Lodz, Poland. Blood samples were collected by qualified medical personnel from healthy male volunteers aged 18–35 years. Eligibility was based on normal laboratory parameters and the absence of a medical history of serious, recurrent, or chronic diseases affecting major organ systems, the coagulation system or other hematological conditions, as well as infectious diseases. Individuals who had undergone surgery or procedures involving disruption of the skin within the preceding four months were excluded. Additional exclusion criteria included smoking, excessive alcohol consumption, and an unbalanced diet. Volunteers who had received vaccinations, antibiotic therapy, or desensitization treatment within the preceding two months were also excluded. The study complied with the Declaration of Helsinki and was approved by the Research Ethics Committee of the Medical University of Lodz (RNN/15/23/KE).

To assess TNF levels, isolated neutrophils (suspended in RPMI 1640 medium supplemented with 10% fetal calf serum and a 1.0% penicillin–streptomycin mixture) were seeded onto 96-well culture plates (2.12 × 10^6^ cells/well). Prior to the analyses, the extracts were dissolved in RPMI 1640 medium. The first step of the experimental procedure involved a 0.5 h pre-incubation of the cells with the test extracts or positive control. Following pre-incubation (conducted in a laboratory incubator with a CO_2_ atmosphere at 37 °C and 95% humidity), a pro-inflammatory response was induced in the neutrophils by adding a lipopolysaccharide (LPS, *E. coli* O111:B4) solution to a final concentration of 0.1 µg/mL. Subsequently, the neutrophils were cultured for 24 h. The total volume of the mixture in the well was 1000 μL. After this step, microplates were centrifuged to obtain supernatants for further analyses. TNF-α amounts in the supernatants were quantified using enzyme-linked immunosorbent assay (ELISA) according to the manufacturer’s instructions (BD Biosciences, San Diego, CA, USA).

Inhibition of ROS production by *f*MLP (*N*-formyl-methionyl-leucyl-phenyl-alanine)-stimulated neutrophils was measured using a luminol-amplified chemiluminescence assay. The isolated neutrophils (suspended in HBSS) were seeded into 96-well microplates (3.5 × 10^5^ cells/well). At the beginning of the tests, a 15 min pre-incubation (in a laboratory CO_2_ incubator at 37 °C and 95% humidity) of cells with the examined extracts or positive control was performed. The ROS production was initiated with *f*-MLP (1.5 µg/mL). Then, luminol (100 µg/mL) was added as a chemiluminescent probe. Prior to the analyses, the extracts were dissolved in an Hanks’ Balanced Salt Solution (HBSS). The total volume of the mixture in the well was 200 μL.

Neutrophil elastase (ELA-2) release from stimulated cells was assessed spectrophotometrically by measuring *p*-nitroaniline generated from *N*-succinyl-alanine-alanine-valine-*p*-nitroanilide (SAAVNA). The isolated neutrophils (suspended in HBSS buffer) were seeded into 96-well microplates (4.0 × 10^6^ cells/well). Prior to the analyses, the extracts were dissolved in HBSS. The experimental procedure started with a 15 min pre-incubation of cells with the examined extracts or positive control. After the pre-incubation (in a laboratory CO_2_ incubator at 37 °C and 95% humidity), the response of neutrophils was induced by adding the *f*-MLP and cytochalasin B solution (final concentrations of 1.0 and 0.5 µg/mL, respectively), and the cells were incubated for 30 min. Then, microplates were centrifuged to obtain supernatants, to which SAAVNA was added as a substrate for ELA-2. The total volume of the mixture in the well was 150 μL. The liberated *p*-nitroaniline was measured at 412 nm using a microplate reader.

ROS, ELA-2 and TNF-α secretion were expressed as the percentage of released markers compared to untreated control samples. Quercetin and dexamethasone were used as positive controls. Extracts were tested at concentrations of 5–100 μg/mL. Measurements were performed in 96-well plates using a SYNERGY 4 microplate reader (BioTek, Winooski, VT, USA). In the tests in the human neutrophil model, control samples were prepared from cells not treated with extracts and/or stimulating factors. In addition, unstimulated control samples containing human neutrophils, luminol, and the tested extracts, but without the addition of an exogenous stimulus, were included to assess the effect of the extracts on basal ROS-associated chemiluminescence in neutrophils. In addition, control samples containing human neutrophils and the tested extracts, but without the addition of an exogenous stimulus, were included in the ELA-2 assay to assess the potential contribution of the extracts to the assay background and the measured signal. However, additional controls using the extracts in the absence of neutrophils or elastase would be useful to further assess whether the extracts may influence the assay, for example through interactions with the substrate. In addition, experiments using purified elastase could help determine whether the extracts directly affect elastase enzymatic activity. These complementary experiments would provide further insight into the mechanisms contributing to the observed changes in ELA-2.

Cellular biocompatibility of the extracts with neutrophils was evaluated by flow cytometry (BD FACSCalibur, BD Biosciences, San Jose, CA, USA) by analyzing cytotoxicity using propidium iodide staining, with Triton X-100 as a positive control [60]. Analyses were performed after 24 h of incubation with neutrophils.

The experiments using human neutrophils were performed using cells obtained from five independent donors. For each donor, each of the two extracts was evaluated at three concentrations, while the positive control was evaluated at two concentrations. Each experimental condition was independently performed in triplicate for each donor. Cells obtained from five independent donors were analyzed separately and were not pooled.

### 3.7. Scavenging Activity Against Multiple Oxidants in Cell-Free Models

The studies were conducted using modified in vitro spectrophotometric methods as described in previous publications [17,67]. Hydroxyl radical (HO^•^) scavenging analysis was performed by spectrophotometrically determining the concentration of colored hydroxy derivatives of salicylic acid formed in the presence of HO^•^ generated in the Fenton reaction. Superoxide (O_2_^•−^) scavenging was assessed based on the reduction in nitro blue tetrazolium by O_2_^•−^, which is produced from xanthine through xanthine oxidase. During this reaction, nitro blue tetrazolium formazan is formed, resulting in a color change in the solution from yellow to blue. The hydrogen peroxide (H_2_O_2_) reduction assay involved spectrophotometric monitoring of quinine imine, the colored product generated from the reaction between H_2_O_2_, 4-aminoantipyrine, and phenol in the presence of horseradish peroxidase. For each extract, three independent sample preparations were analyzed, each using a serial dilution of at least five concentrations measured in triplicate. An individual half-maximal scavenging capacity (SC_50_) value was calculated for each preparation, and the three values were used to calculate the mean and SD.

### 3.8. Statistical Analyses

All samples were assayed in replicates. Results are presented as mean values ± SD (standard deviation) or ± SE (standard error) for the specified number of experiments. Normality of the distribution of the results was verified using the Shapiro–Wilk test, and the homogeneity of variances using the Levene’s test. Statistical significance was determined using one-way analysis of variance (ANOVA) or one-way repeated-measures ANOVA, as appropriate to the experimental design. One-way ANOVA was used for comparisons involving independent experimental groups, whereas repeated-measures ANOVA was applied when the same biological samples/donors were analyzed with different extracts/positive controls or their concentrations. Post hoc Tukey’s test was used for multiple pairwise comparisons, whereas Dunnett’s test was applied for comparisons with a designated control group. Calculations were performed using Statistica 13.1 PL (StatSoft, Krakow, Poland). A significance level of 0.05 was used for all statistical analyses.

## 4. Conclusions

The results indicate that *P. serotina* flowers are a source of polyphenolic compounds with biological activity. Their antioxidant and anti-inflammatory properties, along with a favorable secondary metabolite profile, underscore their health-promoting potential. The study explains some of the cellular mechanisms underlying the observed effects. However, further research is necessary to verify the involvement of other potential molecular targets and mechanisms and to clarify their significance for the biological effects. Consequently, additional studies involving animal models and clinical trials are required to confirm the efficacy and safety of the analyzed extracts. Given the potential variability in composition and activity, which may depend on factors such as the location of plant material collection, further studies utilizing independent collections are also necessary to assess the repeatability of the observed properties. Another important direction for future research is the development of suitable formulations, such as nano- or microcapsules, to improve the release, bioavailability, and efficacy of the bioactive substances. Despite these limitations, the present findings provide a valuable foundation for further investigation of *P. serotina* flowers as a source of bioactive compounds potentially relevant to cardiovascular health.

## Figures and Tables

**Figure 1:**
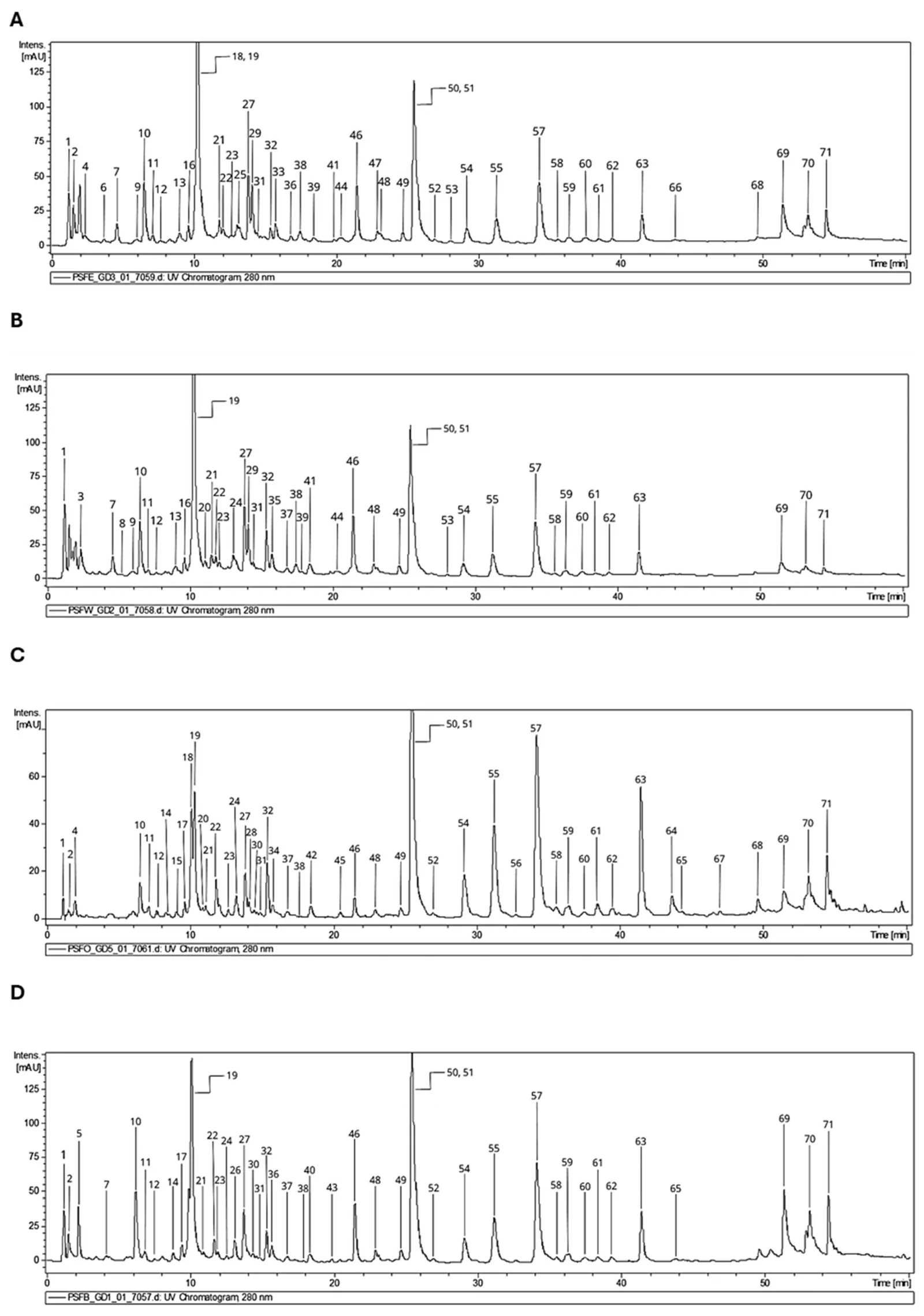
Representative UHPLC chromatograms at 280 nm of (**A**) 70% ethanol–water, (**B**) water, (**C**) ethyl acetate and (**D**) *n*-butanol extracts of *P. serotina* flowers. Peak numbers refer to those implemented in Table 1.

**Figure 2:**
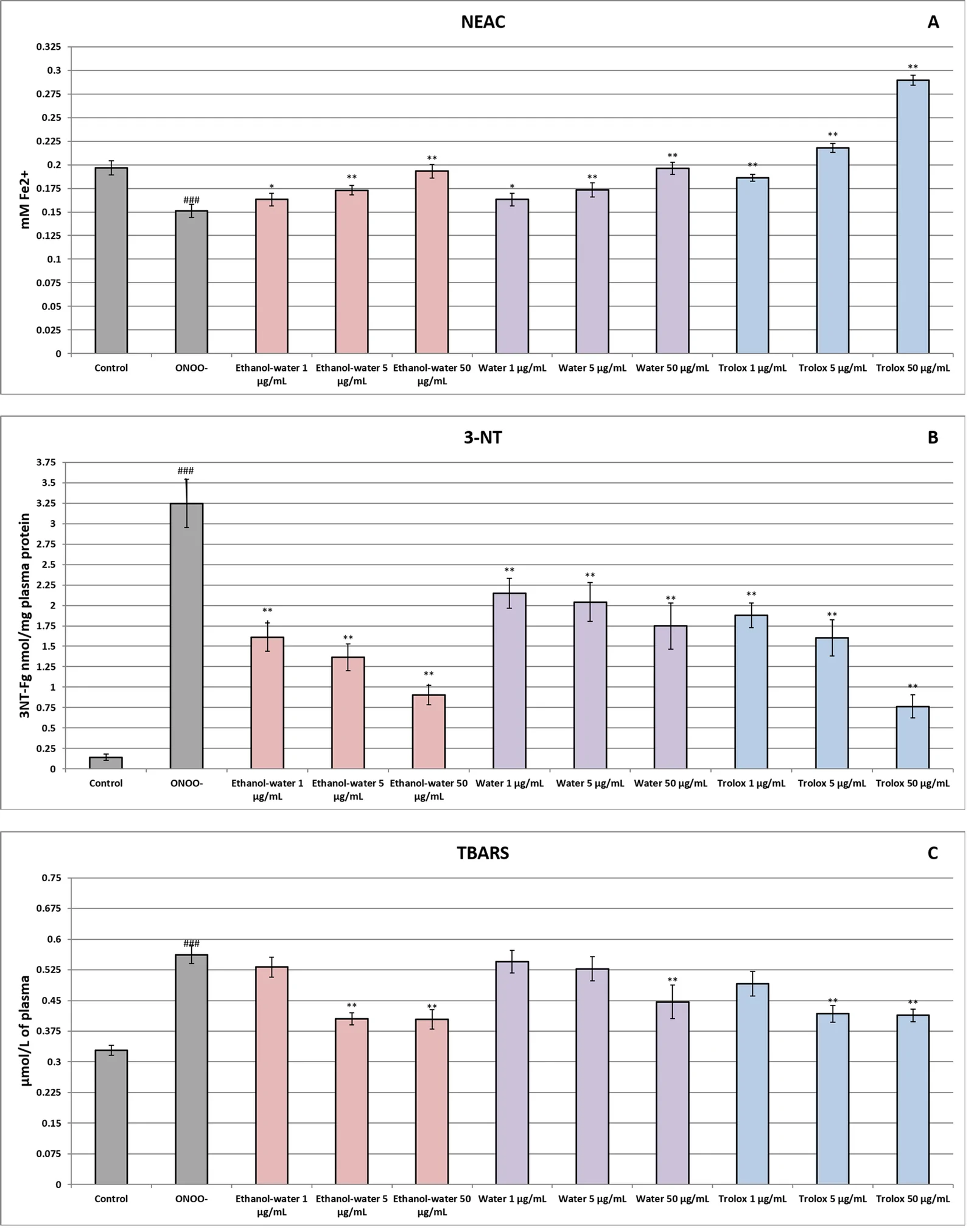
The protective effects of extracts at 1–50 µg/mL on human plasma under oxidative stress: (**A**) effects on the non-enzymatic antioxidant capacity (NEAC) of plasma (assessed by FRAP test); (**B**) influence on the nitration of tyrosine residues in plasma proteins and formation of 3-nitrotyrosine (3-NT); (**C**) impact on the lipid peroxidation and levels of thiobarbituric acid-reactive substances (TBARS). Positive control: Trolox^®^ at 1–50 µg/mL. Data are expressed as mean values ± SE (FRAP: *n* = 8; NEAC and 3-NT: *n* = 9). Statistical significance: ^###^ *p* < 0.001 for control plasma vs. ONOO^−^-treated plasma in the absence of the studied substances; * *p* < 0.05 and ** *p* < 0.01, for ONOO^−^-treated plasma in the presence of the examined extracts and positive control (1–50 μg/mL) vs. ONOO^−^-treated plasma in the absence of the studied substances.

**Figure 3:**
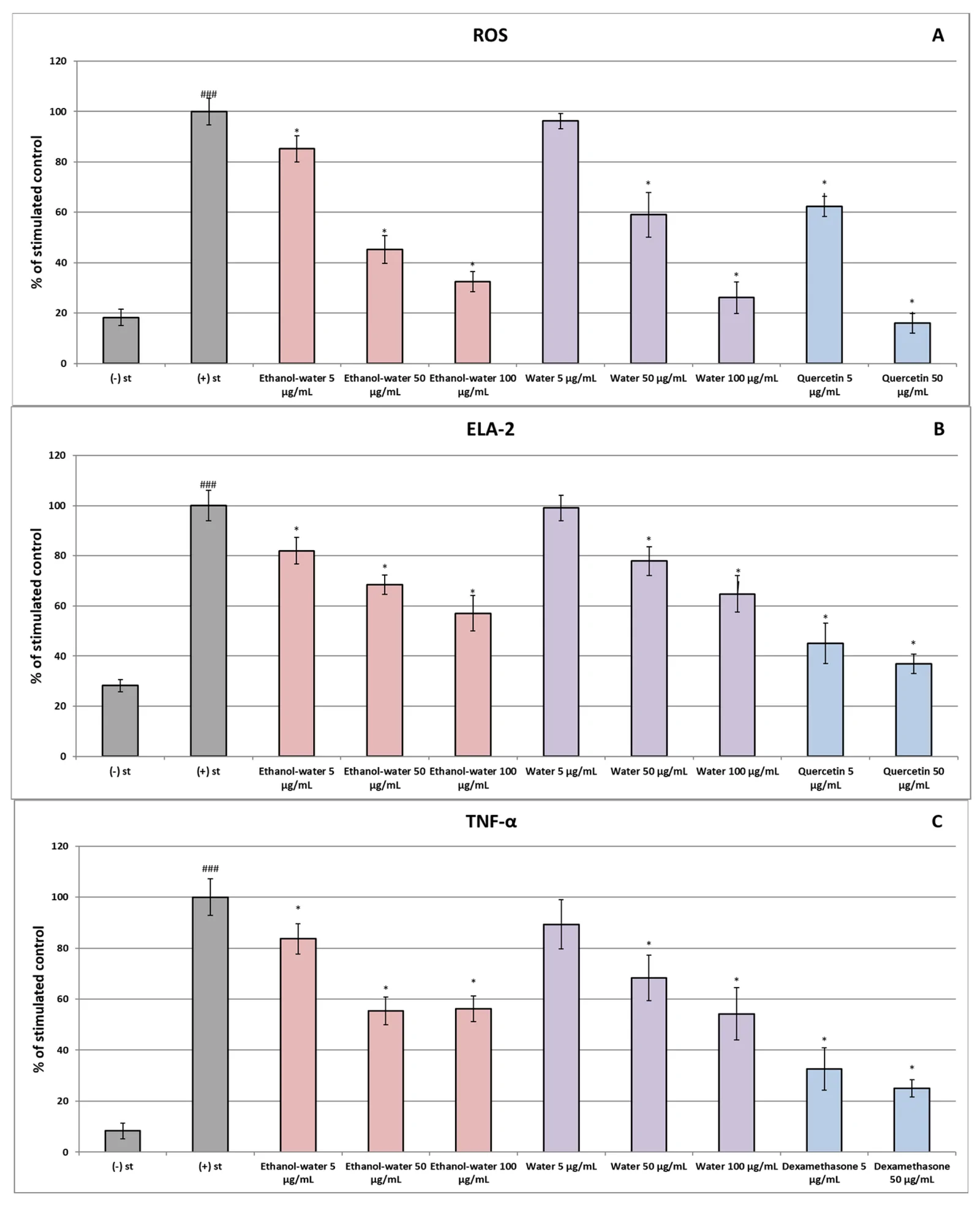
Effect of flowers extracts (5–100 µg/mL) and positive controls (5–50 µg/mL) on the level of (**A**) ROS, (**B**) ELA-2, and (**C**) TNF-α in human neutrophils. Positive controls: quercetin (ROS, ELA-2) and dexamethasone (TNF-α). (−) st, non-stimulated control without extract/positive control; (+) st, stimulated control without extract/positive control. Data expressed as means ± SD of three independent experiments performed with cells isolated from five independent donors. Statistical significance: ### *p* < 0.001 compared to the non-stimulated control; * *p* < 0.05 compared with the stimulated control.

**Table 2:** Content of the polyphenols in the tested extracts (mg/g dw).

	Ethanol–Water Extract	Water Extract	Ethyl Acetate Extract	*n*-Butanol Extract
**Total contents:**				
TPC (GAE)	144.97 ± 2.04 ^a^	119.05 ± 3.39 ^b^	120.39 ± 1.12 ^b^	124.28 ± 4.62 ^b^
TPAC (PB2)	81.08 ± 3.12 ^a^	46.37 ± 2.20 ^b^	6.16 ± 0.26 ^c^	6.72 ± 0.42 ^c^
TLPC	94.37 ± 2.79 ^b^	83.01 ± 0.67 ^c^	97.81 ± 4.74 ^b^	109.09 ± 0.34 ^a^
TFC	54.97 ± 1.66 ^b^	49.72 ± 0.63 ^b^	89.84 ± 4.38 ^a^	89.24 ± 0.89 ^a^
TFAC	9.04 ± 0.30 ^a^	4.95 ± 0.05 ^b^	n.d.	n.d.
TPHAC	29.44 ± 0.86 ^a^	27.64 ± 0.12 ^a^	7.97 ± 0.33 ^c^	19.85 ± 0.55 ^b^
**Individual compounds:**				
Avicularin (Quercetin 3-*O*-α-L-arabinofuranoside) (**57**)	12.35 ± 0.42 ^c^	10.58 ± 0.10 ^c^	28.33 ± 1.04 ^a^	20.24 ± 0.17 ^b^
Hyperoside (Quercetin 3-*O*-β-D-galactoside) (**50**)	23.48 ± 0.71 ^c^	20.60 ± 0.18 ^c^	32.64 ± 2.15 ^b^	39.26 ± 0.34 ^a^
Isorhamentin dihexoside (**46**) *	5.30 ± 0.32 ^a^	6.24 ± 0.31 ^a^	<LOQ	6.12 ± 0.19 ^a^
Juglanin (Kaempferol 3-*O*-α-L-arabinofuranoside) (**63**)	2.46 ± 0.09 ^c^	2.10 ± 0.02 ^c^	9.44 ± 0.25 ^a^	4.48 ± 0.04 ^b^
Kaempferol hexoside (**55**) **	4.56 ± 0.15 ^c^	4.02 ± 0.04 ^c^	10.96 ± 0.59 ^a^	8.18 ± 0.90 ^b^
Reinutrin (Quercetin 3-*O*-α-L-xylopyranoside) (**54**)	2.47 ± 0.08 ^c^	2.06 ± 0.02 ^c^	5.92 ± 0.27 ^a^	4.57 ± 0.04 ^b^
Rutin (Quercetin 3-*O*-(6′′-*O*-α-L-rhamnopyranosyl)-β-D-glucopyranoside) (**51**)	4.34 ± 0.13 ^b^	4.11 ± 0.04 ^b^	2.55 ± 0.23 ^c^	6.39 ± 0.04 ^a^
Epicatechin (**29**)	4.07 ± 0.16 ^a^	2.63 ± 0.05 ^b^	<LOD	<LOD
Procyanidin B1 (**13**)	1.97 ± 0.05 ^a^	1.44 ± 0.01 ^b^	<LOD	<LOD
Proanthocyanidin B type dimer ***	1.40 ± 0.04 ^a^	0.88 ± 0.00 ^b^	<LOD	<LOD
Procyanidin C1 (**39**)	1.59 ± 0.06 ^a^	<LOQ	<LOD	<LOD
Chlorogenic acid (5-*O*-Caffeoylquinic acid) (**19**)	25.44 ± 0.73 ^a^	23.96 ± 0.15 ^a^	5.33 ± 0.27 ^c^	15.74 ± 0.57 ^b^
Cryptochlorogenic acid (4-*O*-Caffeoylquinic acid) (**22**)	2.46 ± 0.12 ^b^	2.18 ± 0.05 ^b^	1.82 ± 0.04 ^c^	2.72 ± 0.01 ^a^
5-*O*-*p*-Coumaroylquinic acid (**27**)	1.54 ± 0.05 ^a^	1.50 ± 0.01 ^a^	0.82 ± 0.03 ^b^	1.39 ± 0.01 ^a^

Results are presented as means ± SD. For each extract, three independent sample preparations were analyzed, with three replicate injections per preparation. The three sample preparations served as replicates for calculating mean ± SD and for statistical analysis. TPC, total phenolic content in gallic acid equivalents (GAE) quantified spectrophotometrically using the Folin–Ciocalteu assay; TPAC, total proanthocyanidin content in procyanidin B2 (PB2) equivalents quantified spectrophotometrically using the *n*-butanol-HCl assay; TLPC, total low molecular mass phenolic content calculated as the sum of individual compounds quantified by RP-HPLC-PDA; TFC, total flavonol content calculated as the sum of individual compounds quantified by RP-HPLC-PDA; TFAC, total low molecular mass flavan-3-ol content calculated as the sum of individual compounds quantified by RP-HPLC-PDA; TPHAC, total phenolic acid content, calculated as the sum of individual compounds quantified by RP-HPLC-PDA; * tentatively identified by LC-MS, quantified using the calibration curve of isorhamnetin 3,7-di-*O*-β-D-glucopyranoside and therefore expressed as isorhamnetin 3,7-di-*O*-β-D-glucopyranoside equivalents; ** tentatively identified by LC-MS, quantified using the calibration curve of juglanin and therefore expressed as juglanin equivalents; *** tentatively identified by LC-MS, quantified using the calibration curve of PB1 and therefore expressed as PB1 equivalents; below the limits of quantitation (LOQ) or detection (LOD); n.d., not detected; dw, dry weight of extract. Values in each row labeled with different letters (a–c) are significantly different at *p* < 0.05. Because only a single extraction process was performed for each solvent, statistical significance refers differences between specific extracts analyzed in this experiment.

**Table 3:** Scavenging activity (SC_50_) towards multiple oxidants generated in cell-free in vitro systems.

	Ethanol—Water Extract	Water Extract	Ethyl Acetate Extract	*n*-Butanol Extract	Trolox^®^
**Target factor**					
HO^•^	145.22 ± 2.04 ^b^	121.09 ± 1.21 ^a^	302.86 ± 29.05 ^d^	226.31 ± 4.67 ^c^	131.67 ± 3.16 ^a, b^
O_2_^•−^	13.02 ± 0.59 ^a^	16.55 ± 1.22 ^a^	23.82 ± 0.32 ^b^	46.64 ± 4.42 ^c^	142.01 ± 5.03 ^d^
H_2_O_2_	111.59 ± 4.59 ^b^	141.47 ± 3.40 ^c^	134.96 ± 6.58 ^c^	152.96 ± 1.25 ^d^	18.10 ± 1.07 ^a^

SC_50_, half scavenging concentration (amount required to reduce the initial oxidant concentration by 50%) calculated in µg/mL. Data are given as means of three replicates ± SD. Values in each row labeled with different letters (a–d) are significantly different with *p* < 0.05. Because only a single extraction process was performed for each solvent, statistical significance refers differences between specific extracts analyzed in this experiment.

**Table 4:** Extraction conditions and yields of *P. serotina* flower extracts.

Solvent	Ethanol–Water (7:3, *v*/*v*)	Water	*n*-Butanol	Ethyl Acetate
**Mass of the plant material [g]**	6.00	6.03	12.0	40.0
**Volume of chloroform [mL]**	100	100	200	650
**1st extraction—volume of solvent [mL]**	150	150	300	1000
**2nd extraction—volume of solvent [mL]**	150	150	300	1000
**3rd extraction—volume of solvent [mL]**	150	150	300	1000
**Mass of the extract [g]**	2.31	2.11	1.73	1.12
**Yield [%]**	38.5	35.0	14.4	2.80

**Table 5:** Selected analytical performance characteristics of the HPLC-PDA quantitative method.

Analyte	Standard Purity	λ (nm)	Regression (Linear Model)		R^2^	Calibration Range (μg/mL)	*F*-Test	LOD (μg/mL)	LOQ (μg/mL)	Repeatability (RSD%)
			Equation	*n*						
Avicularin ^B^	≥98.0	350	y = 9140.9x + 1288.6	6	1.0000	0.99–49.6	467415.1	0.151	0.457	0.170
Chlorogenic acid ^A^	≥99.0	325	y = 14521x + 59.566	6	0.9999	1.18–44.1	40924.81	0.765	2.319	1.923
*p*-Coumaric acid ^A^	≥99.0	310	y = 36279x − 5805.8	6	0.9999	1.33–66.3	64086.43	0.067	0.204	0.068
Cryptochlorogenic acid ^A^	≥99.0	325	y = 11114x + 18664	6	0.9989	1.05–52.5	3328.493	0.337	1.021	1.408
Epicatechin ^A^	≥99.0	280	y = 4099.2x − 6579.5	5	0.9991	6.72–67.2	3537.672	1.942	5.886	0.866
Hyperoside ^A^	≥99.0	350	y = 9698x − 6.7089	6	1.0000	1.14–57.2	87215.73	0.303	0.919	0.178
Juglanin ^B^	≥98.0	350	y = 10273x − 1733.2	6	0.9999	1.20–60.0	60377.96	0.522	1.583	0.445
Procyanidin B1 ^A^	≥96.0	280	y = 3184.7x − 985.83	5	0.9993	4.17–41.7	16184.70	0.783	2.373	0.690
Procyanidin C1 ^A^	≥96.0	280	y = 3060.4x − 2452.9	5	0.9999	5.09–50.9	38588.72	1.076	3.261	1.166
Reinutrin ^B^	≥98.0	350	y = 11532x − 2256.2	6	0.9999	1.22–61.1	1988.707	0.262	0.795	0.420
Rutin ^A^	≥97.0	350	y = 7363.7x − 922.95	6	0.9999	1.37–68.7	35407.09	0.267	0.808	0.937

^A^, compounds obtained from PhytoLab GmbH & Co. KG (Vestenbergsgreuth, Germany); ^B^, compounds isolated previously in our laboratory from the flowers and leaves of *P.spinosa* [62,63,64]; λ, detection wavelength; y, peak area; x, concentration of standard in (μg/mL); *n*, number of concentration levels (data points) used for construction of the regression equation; *F*-test, value of the statistical Fisher variance ratio for the experimental data.

## Data Availability

The original contributions presented in this study are included in the article. Further inquiries can be directed to the corresponding author.

## Abbreviations

The following abbreviations are used in this manuscript:

3-NT	3-Nitrotyrosine
3-NT-FG	Nitrated fibrinogen
CVDs	Cardiovascular diseases
ELA-2	Enzyme elastase-2
ELISA	Enzyme-linked immunosorbent assay
*f*MLP	*N*-Formyl-methionyl-leucyl-phenyl-alanine
GAE	Gallic acid equivalents
H_2_O_2_	Hydrogen peroxide
HO^•^	Hydroxyl radical
LDLs	Low-density lipoproteins
NEAC	Non-enzymatic antioxidant capacity of human plasma
NF-κB	Nuclear factor kappa-light-chain-enhancer of activated B cells
NLR	Neutrophil-to-lymphocyte ratio
NO	Nitric oxide
NRF2	Nuclear factor erythroid 2-related factor 2
O_2_^•−^	Superoxide
ONOO^–^	Peroxynitrite
ROS	Reactive oxygen species
SAAVNA	*N*-succinyl-alanine-alanine-valine-*p*-nitroanilide
SC_50_	Half maximal scavenging capacity
SOD	Superoxide dismutase
TBARS	Thiobarbituric acid-reactive substances
TNF-α	Tumor necrosis factor α
TFAC	Total content of low-molecular-weight flavan-3-ols
TFC	Total content of flavonols
TLPC	Total content of low molecular mass phenolics
TPAC	Total content of proanthocyanidins
TPC	Total content of polyphenols
TPHAC	Total content of phenolic acids
